# Monitoring extracellular pH, oxygen, and dopamine during reward delivery in the striatum of primates

**DOI:** 10.3389/fnbeh.2012.00036

**Published:** 2012-07-05

**Authors:** Jennifer L. Ariansen, Michael L. A. V. Heien, Andre Hermans, Paul E. M. Phillips, Istvan Hernadi, Maria A. Bermudez, Wolfram Schultz, R. Mark Wightman

**Affiliations:** ^1^Department of Chemistry and Neurobiology Curriculum, University of North Carolina at Chapel Hill, Chapel HillNC, USA; ^2^Department of Physiology, Development and Neuroscience, University of CambridgeCambridge, UK

**Keywords:** cyclic voltammetry, reward, primate, cerebral blood flow, oxygen, pH

## Abstract

Dopamine projections that extend from the ventral tegmental area to the striatum have been implicated in the biological basis for behaviors associated with reward and addiction. Until recently, it has been difficult to evaluate the complex balance of energy utilization and neural activity in the striatum. Many techniques such as electrophysiology, functional magnetic resonance imaging (fMRI), and fast-scan cyclic voltammetry have been employed to monitor these neurochemical and neurophysiological changes. In this brain region, physiological responses to cues and rewards cause local, transient pH changes. Oxygen and pH are coupled in the brain through a complex system of blood flow and metabolism as a result of transient neural activity. Indeed, this balance is at the heart of imaging studies such as fMRI. To this end, we measured pH and O_2_ changes with fast-scan cyclic voltammetry in the striatum as indices of changes in metabolism and blood flow *in vivo* in three *Macaca mulatta* monkeys during reward-based behaviors. Specifically, the animals were presented with Pavlovian conditioned cues that predicted different probabilities of liquid reward. They also received free reward without predictive cues. The primary detected change consisted of pH shifts in the striatal extracellular environment following the reward predicting cues or the free reward. We observed three types of cue responses that consisted of purely basic pH shifts, basic pH shifts followed by acidic pH shifts, and purely acidic pH shifts. These responses increased with reward probability, but were not significantly different from each other. The pH changes were accompanied by increases in extracellular O_2_. The changes in pH and extracellular O_2_ are consistent with current theories of metabolism and blood flow. However, they were of sufficient magnitude that they masked dopamine changes in the majority of cases. The findings suggest a role of these chemical responses in neuronal reward processing.

## Introduction

Neural events that occur following the presentation of a reward or a cue that predicts reward have been studied extensively. A prime focus of this research has been on the mesolimbic dopamine system (Dichiara and Imperato, 1988; Schultz et al., 1997; Phillips et al., 2003; Wise, 2004; Pan et al., 2005; Ikemoto, 2007; Roitman et al., 2008; Owesson-White et al., 2009). Methods for evaluating neural function in reward processing include electrophysiology, electrochemistry, and functional magnetic resonance imaging (fMRI). Electrophysiological data have shown that dopamine neurons originating in the ventral tegmental area are activated by unexpected rewards and cues that predict rewards (Fiorillo et al., 2003; Tobler et al., 2005). Blood oxygen level dependent (BOLD) fMRI activations have been demonstrated in the human dorsal and ventral striatum and the prefrontal cortex, which implicates these brain regions in reward-related behaviors (O'doherty, 2004; Tobler et al., 2007, 2009; Haber and Knutson, 2010). The BOLD signal arises from the level of blood oxygenation, which is governed by the coupling of neural activity and blood flow in the brain. In the rat, fast-scan cyclic voltammetry allows electrochemical recordings with a carbon-fiber microelectrode that provides a view of dopamine concentration changes as well as extracellular O_2_ and pH fluctuations with subsecond temporal resolution (Venton et al., 2003; Takmakov et al., 2010a). These simultaneous measurements of dopamine, O_2_, and pH provide a method to probe the relationships between increased neural activity and blood flow. While electrophysiology and BOLD recordings have been made in primates, electrochemistry techniques have only been used rarely in primates (Earl et al., 1998; Kishida et al., 2011; Yoshimi et al., 2011).

Unraveling the relationships between the chemical changes arising from neural activity and the subsequent physiological responses during reward-based behaviors is being explored in rodents (Cheer et al., 2006). Current theory suggests that increased neural activity in response to rewards or reward-predicting cues increases blood flow, resulting in increased O_2_ levels and clearance of CO_2_ that causes alkaline pH shifts. In balance with this mechanism, increases in metabolism cause increases in CO_2_ and lactic acid concentration that can result in an acidic pH shift (Kaila, 1998; Chesler, 2003; Venton et al., 2003). Concurrent voltammetric studies of extracellular dopamine in the primate striatum in brain slices (Cragg et al., 2000, 2002) and in the anesthetized preparation (Earl et al., 1998) have revealed that dopamine levels are controlled similarly as in rodents (Robinson et al., 2008), in that evoked dopamine release is frequency dependent and removed by uptake. In fact, one recent study demonstrated catecholamine release in the caudate of primates during Pavlovian reward conditioning, but the data was collected using amperometry, so chemical validation of the signal was not conclusive (Yoshimi et al., 2011). However, not one of these reports addressed changes in extracellular pH or oxygen levels. So, in the current study, we employed fast scan cyclic voltammetry to investigate the roles of oxygen, pH, and dopamine at the synaptic level during reward related behavior in the primate striatum. According to the model, we hypothesized that neural activity in response to cues that predict rewards or the rewards themselves would induce an O_2_ change concomitant to a pH change, offering new insight into downstream dynamics of neural activity that run in conjunction with known dopaminergic changes. While such changes occurred, they were of sufficient amplitude to mask the dopamine changes in the majority of cases.

## Materials and methods

### Chemicals

Chemicals were purchased from Sigma-Aldrich (St. Louis, MO) and used as received. For flow injection experiments phosphate buffered saline (150 mM NaCl, 10 mM Na_2_HPO_4_, 1.2 mM CaCl_2_) was used. Stock solutions of dopamine were prepared in 0.1 M HCl, and were diluted to the desired concentration on the day of use. Tungsten etching was performed in 1.0 N NaOH solution, saturated with NaNO_2_. The diazonium salt, 4-sulfobenzenediazonuim tetrafluoroborate, was synthesized as previously described (Hermans et al., 2006).

### Electrodes

The carbon-fiber microelectrodes were fabricated from two types of carbon fibers: 12 μm diameter (Thornel P55, Amoco, Greenville, SC) and 33 μm diameter carbon fibers (Textron Systems Division, Wilmington, MA) with a tungsten wire (125 μm diameter, 15 cm length, Advent research Materials, Oxford, England) as support. Tungsten wires were etched to a conical tip and were cleaned by applying 4.0 V in electrocleaning solution (Grobet USA, Carlstadt, NJ) (Hermans and Wightman, 2006). Carbon-fibers were attached along the whole length of the tungsten wire with conductive silver epoxy (Epo-tek, Billerica, MA) extending from the tip of the tungsten wire approximately 2 cm. Afterwards, the tungsten wire and carbon fiber were inserted into a glass capillary and pulled in a horizontal electrode puller. After the heating element reached a temperature sufficient to soften the glass, the movable capillary holder, which held the non-etched end of the tungsten wire, was slowly pulled resulting in a thin (~5 μm) glass layer over the whole length of the tungsten wire.

The assembly was inspected under a microscope to ensure a smooth transition of the glass over the tip of the tungsten wire and the carbon fiber. The carbon fiber and the glass were cut with a scalpel blade approximately 250 μm from the end of the tungsten tip. Another insulating layer was applied to the electrode tips with Epoxylite insulation (The Epoxylite Corporation, St. Louis) at 40°C for 1 min and then slowly withdrawn (1 mm/10 s). The electrodes were cured for 8 h at 80°C. The electrodes were subsequently polished at a 25° angle on a micropipette beveller (Sutter instrument, Novato, CA), and were cycled in PBS buffer from –0.4 V to 1.3 V vs. Ag/AgCl to activate the electrode (Takmakov et al., 2010b). Following the electrochemical pretreatment P-55 microelectrodes with 12 μm fibers described above were coated with 4-sulfobenzene by applying a potential of –1 V vs Ag/AgCl to the electrode for 5 min in a 3 mM solution of 4-sulfobenzenediazonium tetrafluoroborate dissolved in 0.1 M HCl (Hermans et al., 2006). Electrodes manufactured from 33 μm diameter fiber did not undergo this treatment. Both types of electrodes were dip-coated with Nafion (Kawagoe et al., 1993), and the response to acidic and basic pH shifts and known dopamine concentrations was assessed with a flow-injection apparatus.

### Flow-injection apparatus

Prior to *in vivo* experimentation, each electrode was tested in known concentrations of dopamine and acidic and basic pH shifts via a flow-injection apparatus. The electrode was positioned at the outlet of a 6-port rotary valve (Rheodyne model 5041 valve). The analyte was loaded into an injection loop and delivered to the surface of the electrode following manual switching of the 6-port valve. The flow rate, driven by gravity, was approximately 2 ml/s. For oxygen calibrations, each electrode was tested in a similar manner as previously described (Zimmerman and Wightman, 1991). To maintain constant oxygen concentrations, glass syringes were used and the flow injection system was fitted with PEEK tubing to limit unwanted entry or loss of oxygen.

### *In vivo* recordings

The experimental design is similar to experiments reported previously (Tobler et al., 2003, 2005; Bermudez and Schultz, 2010). Recordings were made in three *Macaca mulatta* monkeys that were mildly fluid deprived (~200 mL liquid/day). All procedures were performed in Cambridge (UK), complied with the UK Animal Protection Law, and were supervised by the UK Home Office. Voltammetric recordings were made in the striatum (caudate and putamen). The reward was a sweetened liquid delivered by a computer-controlled solenoid valve from a spout at the animal's mouth in fixed quantities of 0.2 ml.

Animals were submitted to Pavlovian conditioning with several visual stimuli that predicted the probability (*p* = 0.05, 0.50, or 0.95) of subsequent reward delivery. Each trial was initiated by a central fixation spot (CF) shown on a computer screen approximately 500 mm from the animal's face. The monkey was required to touch a resting key within 500 ms of CF appearance. Three different visual cues with similar physical salience served as the conditioned stimuli (CS) and were presented between 1.5 and 2 s after key touch. Another 1.5 s later the color of the CF changed from red to green, prompting the monkey to release the key. The reward (unconditioned stimulus, US) was delivered to the animal's mouth 1 s after the key was released. The computer screen went blank 0.5 s after reward delivery. The next trial started 3.5 ± 0.5 s later. At each striatal recording location approximately 90 behavioral trials were performed, with approximately 30 trials for each reward probability (*p* = 0.05, *p* = 0.50, and *p* = 0.95, alternating pseudorandomly). Thus only 1 or 2 trials were rewarded in *p* = 0.05 trials, and only 1 or 2 trials went unrewarded in *p* = 0.95 trials.

Unpredicted, free reward was delivered in separate blocks of 15 trials. The time between each free reward delivery was 4 s plus an interval drawn from a pseudoexponential distribution with a mean of 5 s that was truncated at 15 s. Thus, inter-reward intervals ranged from 4 to 19 s. The time between each block of free reward trials, thus, was at least 30 mins. The free reward trial was performed after completion of the 90 conditioned reward trials. Electrode responses on the 15 similar conditions at each location were averaged around the time of reward delivery.

### Data acquisition and analysis

Recordings were conducted in 26 striatal recording sites in animal 1, 22 striatal locations in animal 2, and 3 striatal locations in animal 3. The recording locations were confirmed by histological examination of stereotaxically oriented coronal brain sections.

Fast-scan cyclic voltammograms were acquired and analyzed using locally constructed hardware and software written in LabVIEW (National Instruments, Austin, TX), modified to run on a laptop computer. Triangular excursions were normally from –0.6 V or –0.4 V vs. Ag/AgCl to 1.0 V or 1.4 V at a scan rate of 400 V/s (Heien et al., 2003). For experiments in which O_2_ was measured the waveform began with a scan from 0.0 V to +0.8 V, a reversal to –1.4 V, and then returned to 0.0 V. During measurements, the waveforms were repeated at 10 Hz. The signal was filtered at 10 kHz before being digitized. The behavior was synchronized to the voltammetric recordings with TTL signals at the onset of each event. Cyclic voltammograms were background subtracted. Data collected at the same location and with the same CS (or unpredicted reward) were signal averaged. Color representations were used to visualize the data with the applied potential as ordinate and time as abscissa with the current represented by a non-linear color scale (Heien et al., 2003). In some locations the data were analyzed by principle component regression (Heien et al., 2004).

## Results

### Responses to pH changes, oxygen, and dopamine at carbon-fiber microelectrodes

**Figure 1:**
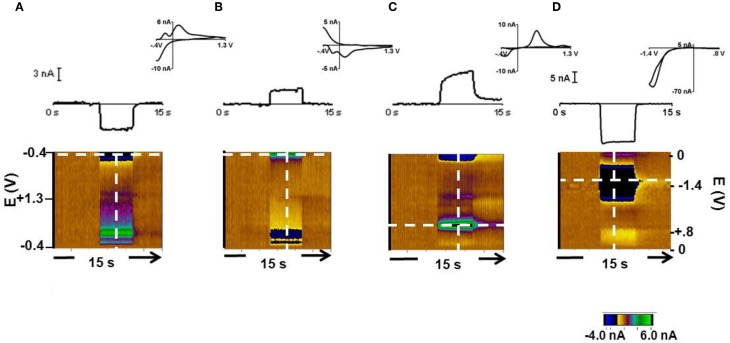
**Cyclic voltammetric responses of pH, dopamine and oxygen with a 12 μm carbon-fiber electrode.** The cyclic voltammograms (top, inset), the current during the 15 s injection at the peak potential of interest (middle) and the color plots are shown. The color plot shows all changes in current (in false color, greens are positive changes, blues are negative changes) at the applied potentials (y-axis) over time (x-axis) (Michael et al., 1998) **(A)**. Response during exposure to a 0.3 pH acidic change. Current was monitored at the Q-peak (−0.3 V on the cathodic scan, white dashed horizontal line) of the triangular waveform −0.4 V to +1.3 V. **(B)** Response during exposure to a 0.3 pH basic change with current measured at the Q peak. **(C)** Response to 2 μM dopamine. Its oxidation occurs at +0.65 V on the anodic scan with the −0.4 V to +1.3 V waveform. **(D)** Response to 24.4 μM O_2_ with a 0.0 V to 0.8 V to −1.4 V to 0.0 V waveform. Oxygen reduction occurs at −1.2 V.

Changes in pH and dopamine can be measured by carbon-fiber microelectrodes with background subtracted cyclic voltammetry (Takmakov et al., 2010a). Representative responses at the 12 μm carbon fiber are shown in Figure 1. In response to 0.3 acidic (Figure 1A) and a 0.3 basic (Figure 1B) pH relative to pH 7.4, the current changed at several potentials as seen in the color plot and cyclic voltammograms. Prior work has shown that the current at −0.3 V on the negative going scan (the Q-peak) is most useful for tracking pH changes (Takmakov et al., 2010a). The response to a 2 μM bolus of dopamine with the same −0.4–1.3 V waveform is shown in Figure 1C. The current at the Q-peak of the acidic and basic pH changes and at the oxidation peak for dopamine are shown in the upper part of Figures 1A,B, and C (obtained at the potentials indicated by the horizontal dotted line on the color plots). The cyclic voltammograms shown for each condition were recorded at the vertical lines in the color plots. The cyclic voltammograms for acidic and basic pH changes are highly comparable to voltammograms reported previously with the same scan parameters (Takmakov et al., 2010a). The sensitivity of these electrodes was 8 ± 3 nA/μM dopamine (*n* = 8 electrodes, ± SEM) and 4.1 ± 0.12 nA/+0.3 pH units and −4.6 ± 0.13 nA/–0.3 pH units at the Q-peak. The detection limit for dopamine, which represents the differential change based on background subtraction, at these electrodes was approximately 15 nM. Finally, the response to 24.4 μM O_2_ with the 0.0 V to 0.8 V to −1.4 V to 0.0 V waveform (Figure 1D) is shown.

The 33 μm electrodes showed similar responses to each condition. The average sensitivity for these electrodes was 13 ± 4 nA/μM for dopamine (*n* = 7 electrodes, ± SEM) and 4.35 ± 0.15 nA/ for pH changes of +0.03 and −0.03 pH units. These electrodes had a detection limit of approximately 25 nM dopamine. The electroactive surface area of the 12 μm electrodes was approximately 0.3 × 10^−5^ cm^2^ and was 2 × 10^−5^ cm^2^ for the 33 μm electrodes. The two electrode configurations were used in this study to evaluate whether electrode diameter affects the measured signals. In fact, similar responses were obtained with each type. For example, the two electrodes showed comparable sensitivities to the Q-peak for oxygen detection.

### *In vivo* pH changes during the presentation of reward predicting cues

**Figure 2:**
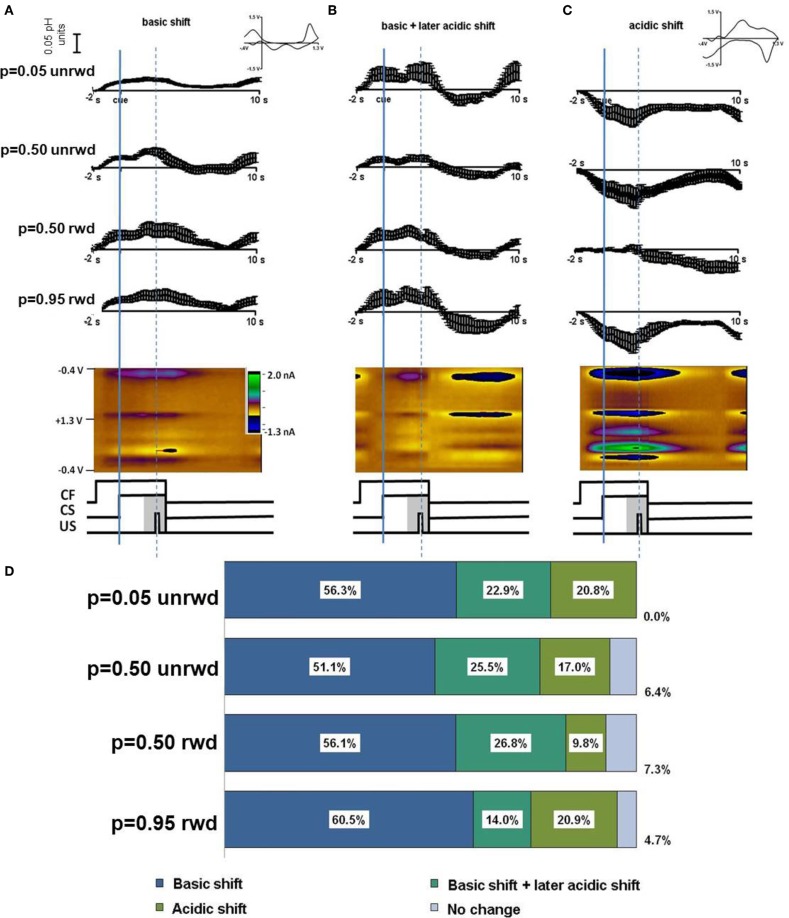
***In vivo* response to cues predicting different probabilities of reward.** Responses are sorted by predicted probability and reward delivered. Averages are presented from Animal 1 and 2, respectively. **(A)** Average current measured at the Q peak and color plot for a basic pH shift following the CF. **(B)** Average current at the Q peak and color plot for a basic pH shift following the CF and a later acidic pH shift. **(C)** An acidic shift and color plot following the CF. For 5% probability only unrewarded trials are shown. For 50% probability trials both unrewarded and rewarded trials are shown. For 95% probability trials only rewarded trials are shown. The cue was initiated at timestamp 0 s and the reward was delivered at the dotted line (in rewarded trials). **(D)** A summary of the percentage of responses by probability cue type. The largest number of responses was a basic shift (blue, *n* = 100 responses overall) a basic shift followed by an acidic shift (turquoise, *n* = 40 responses overall) or a long term acidic shift (green, *n* = 26 responses overall). A small percentage of responses showed no change (purple, *n* = 8 responses overall).

Timing diagrams of the sequence of events during predicted reward are shown in the central panels of Figure 2. We sorted cyclic voltammetric data from each location according to reward probability, *p* = 0.05, *p* = 0.50, or *p* = 0.95 and relative to the occurrence (or lack) of reward delivery. At a single location, the sorted data were averaged around the reward-predicting cue (CS). We did not analyze rewarded trials with probability of *p* = 0.05 or unrewarded trials with *p* = 0.95 because of their infrequent occurrence. Trials with *p* = 0.50 probability of reward were analyzed separately according to the delivery or non-delivery of reward (Figure 2, rows 2 and 3, respectively).

The cyclic voltammograms revealed that basic or acidic pH shifts occurred with all predicted rewards (insets, Figure 2). We observed three types of responses that occurred with all three different reward probabilities. A short term basic pH shift (Figure 2A) was the most frequent. We also observed a short term basic pH shift that was followed by an acidic pH shift (Figure 2B), as well as acidic pH shifts without a basic excursion (Figure 2C). Figure 2D tabulates the percentage of each type of response according to reward probability. The short term basic shifts occurred in 75–83% of the recording locations, regardless of cue probability or rewarded or unrewarded trial outcomes (blue section, Figure 2D). The acidic pH changes following the basic pH change also occurred with every reward probability scenario, occurring in 15–27% of recordings (turquoise section, Figure 2D). Acidic changes not associated with a preceding basic pH shift occurred in approximately 18% of recordings overall (green section, Figure 2D). Only 8% of recordings for any given reward probability showed no measurable pH change (purple section, Figure 2D). Features characteristic of dopamine oxidation were not apparent in these data.

### *In vivo* pH and catecholamine changes in free reward trials

**Figure 3:**
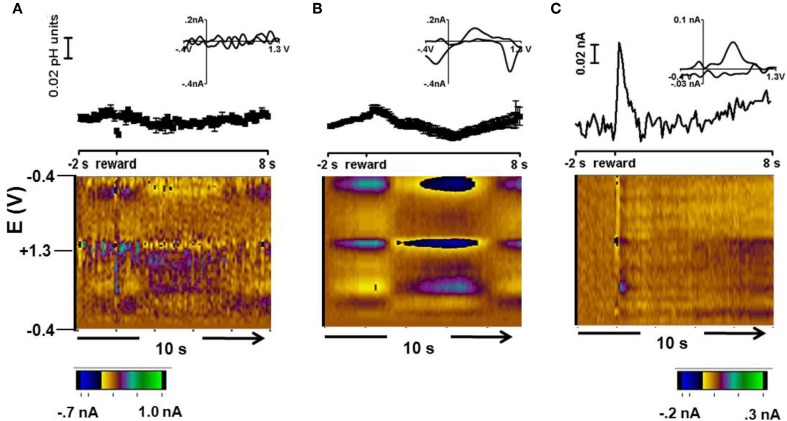
**Free reward response. (A)** Representative cyclic voltammogram (top, inset), concentration vs time (middle), and average color plot at two locations where there was no change following the free reward. **(B)** Representative cyclic voltammogram (top, inset), concentration vs. time (middle), and average color plot representation of the response in about 90% of the recording locations. **(C)** Cyclic voltammogram (top, inset), current vs time (middle), and color plot of a dopamine-like signal in the residual after accounting for pH with principal component analysis.

Figure 3 shows a representative color plot and concentration trace average for 15 trials obtained during free reward trials in Animal 1. These data were averaged to the time of reward presentation. The background was taken from averaged scans between 0.5 s – 1.5 s before reward presentation. Cyclic voltammograms for each type of response are presented in the inset of Figures 3A and B. Overall there were two types of responses: no significant change in pH following free reward in ~10% of recordings (two locations) (Figure 3A) and a long term acidic shift lasting for approximately 5 s following the reward observed in ~90% of recordings (31 locations, Figure 3B).

To resolve a dopaminergic signal from the signals measured during free reward, principal component regression was employed (Heien et al., 2004; Keithley et al., 2009). Here, the signal obtained for the pH shift was used as the only principal component and the resulting residual was examined in the form of a color plot. Interestingly, in the example shown in Figure 3C, an increase in oxidation current occurred at the potential at which dopamine is oxidized immediately after delivery of the reward. The cyclic voltammogram at the maximum response has the features of authentic dopamine recorded in pH 7.4 buffer. The time course of the current traces reveals that the dopamine-like signal increases rapidly following reward delivery and then returns to baseline (Figure 3C, top panel). A similar dopamine-like signal occurred in 14% of the striatal recording locations during free reward trials. A dopamine signal was not recovered in the remaining trials.

**Figure 4:**
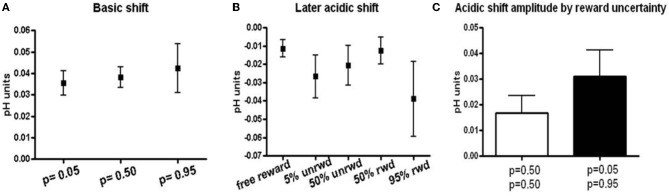
**Basic and acidic pH peak amplitude. (A)** Peak amplitudes for responses that showed a basic pH shift following the cue presentation. Peak amplitudes were taken at approximately 1.5 s after the cue presentation. **(B)** Peak amplitudes for the later acidic pH shift following the basic pH shift. Peak amplitudes were taken between 6–7 s following the cue presentation or at the minima in the case of free reward. **(C)** Peak amplitudes of acidic pH shift sorted according to predicted uncertainty. Rewarded and unrewarded trials following a cue that predicted 50% reward probability are grouped together as they have the same uncertainty. Unrewarded trials following the *p* = 0.05 cue and rewarded trials following the *p* = 0.95 cue are shown in black.

The peak amplitudes of basic pH shifts increased with reward probability. Thus, cues predicting *p* = 0.05 reward probability induced a 0.035 ± 0.006 (*n* = 38 ± SEM) basic pH shift, cues predicting *p* = 0.50 induced a 0.038 ± 0.005 (*n* = 57 ± SEM) basic pH shift, and cues predicting *p* = 0.95 induced a 0.042 ± 0.011 (*n* = 31 ± SEM) basic pH shift (Figure 4A). While the data show a trend for increasing basic shifts with increased reward probability, the three conditions were not significantly different from one another. There was no significant difference or trend in the later acidic shift following a basic shift in trials with reward predicting cues (Figure 4B). Note, that the acidic shift observed following most free rewards also occurred at the time of anticipated reward in the trials with reward predicting cues.

Lastly, we related the long term acidic shift amplitudes to the risk of reward as defined by the standard deviation of the three probability distributions (*p* = 0.05, *p* = 0.50, *p* = 0.95). The *p* = 0.50 reward probability is associated with the highest risk, whereas the *p* = 0.05 and *p* = 0.95 reward probabilities have the lowest risk. There was a trend for lower risk to be associated with larger acidic shift (0.017 ± SEM for *p* = 0.50 probability and 0.031 ± SEM for *p* = 0.05 and *p* = 0.95), although these differences were insignificant (*p* > 0.230, Figure 4C).

### Dopamine-like signal and O_2_ responses

**Figure 5:**
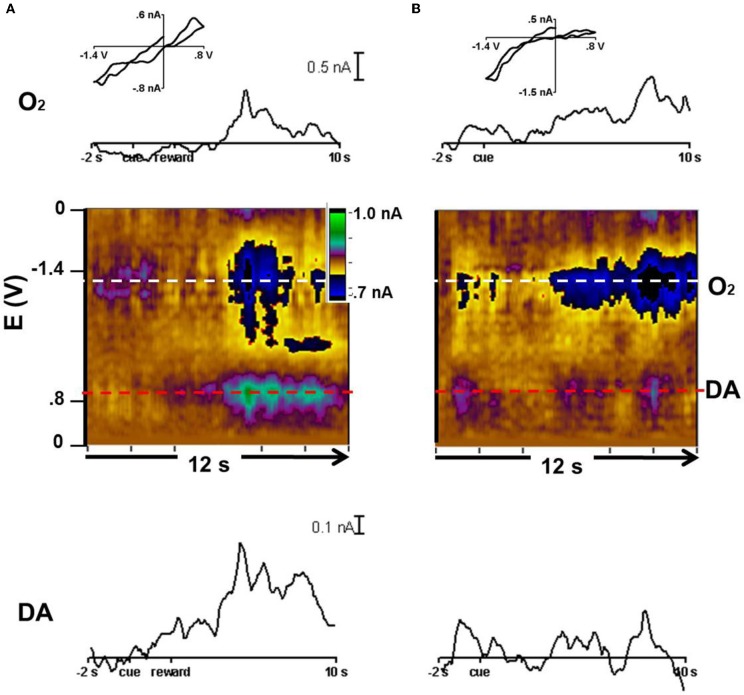
**Dopamine-like signal and oxygen increases in *p* = 0.50 trials.** Current vs. time trace, color plot, and representative cyclic voltammogram for dopamine and O_2_. **(A)** Reward trials averaged to the cue at 0 s. The current vs time trace for O_2_ (horizontal white line in color plot) and dopamine (red in color plot). Below: the average color plot of 22 rewarded trials in which the cue predicted a 50% probability of reward. The cyclic voltammogram (inset) was taken at 6.5 s. **(B)** Current vs time trace for O_2_ (white) and dopamine (red) and average color plot of 14 unrewarded trials in which the cue predicted a 50% probability of reward. The cyclic voltammogram (inset) was taken at 8 s.

In animal 3, we tested the CS predicting reward with probability of *p* = 0.50. The cyclic voltammograms were obtained with waveforms that allowed detection of both dopamine and oxygen. With rewarded trials, O_2_ and the dopamine-like signal increased after reward delivery as revealed by the color plots and the averaged currents associated with each species (Figure 5A). In the unrewarded trials, the O_2_ increase lasted longer than in the rewarded trials and there was a much lower response at the potential where dopamine is oxidized (Figure 5B). Though this response is much smaller compared to the other changes present in these recordings, in both rewarded and unrewarded trial types, an ~0.1nA dopamine-like response occurred at the time of the initial cue. These responses are the average of those obtained in the caudate during three recording days totaling 36 trials.

## Discussion

The aim of the present study was to assess the chemical changes that are accessible to fast-scan cyclic voltammetry and occur in the primate striatum during reward processing. Based on previous work using electrophysiology, fMRI and electrochemistry, we hypothesized that there would be O_2_ and pH changes associated with reward and the cues that predict reward. The direct demonstration of these changes *in vivo* provides new information on the neurochemical processes that occur with neural activity associated with reward-based behaviors. Secondary to that goal, we also hypothesized that there would be dopamine release to the cue and the reward as previously characterized by electrophysiology and electrochemistry (Schultz, 2002; Phillips et al., 2003; Cheer et al., 2006; Day et al., 2007; Roitman et al., 2008; Owesson-White et al., 2009; Jones et al., 2010; Wheeler et al., 2011). In fact, we made two major discoveries. First, in the primate striatum acidic and alkaline pH shifts accompanied by oxygen changes occur during cue and reward presentation. Second, we found that these responses are sufficiently large as to overshadow dopaminergic responses in the majority of cases.

The extracellular O_2_ concentration in the brain is the balance between that consumed by metabolism and that provided by increases in blood flow. In turn, the extracellular pH in the brain is coupled to O_2_ changes because it is dependent on the same processes. The pH of the extracellular fluid is governed by the H_2_CO_3_/HCO^−^_3_ buffering system. The concentration of H_2_CO_3_ is in equilibrium with [CO_2_]. Thus, acidic pH shifts can arise from an increase in [CO_2_] arising from the oxidation of glucose either anaerobically, with the production of lactic acid and carbon dioxide, or aerobically with the production of CO_2_ and water (H_2_O) (Kaila, 1998; Chesler, 2003). Increased blood flow that delivers oxygen to the localized brain region also clears CO_2_, causing an alkaline pH shift (Urbanics et al., 1978). Typically, increased neural activity leads to an increase in blood flow that exceeds the metabolic requirements (Raichle, 1998). For example, Venton and coworkers demonstrated that stimulation of the substantia nigra in the rat induced time-locked basic pH shifts and O_2_ increases that occurred approximately 2 s after the stimulation (Venton et al., 2003). Thus, previous work using fast-scan cyclic voltammetry has revealed the coupled nature of oxygen and pH changes that accompany increased activity of striatal nerve terminals. Though the precise nature of the chemical signaling in the striatum responsible for the increase in blood flow is still to be established and linking relative acidity and oxygen changes in the context of reward learning is considerably more difficult than what we currently know about dopamine and reward learning, the experiments in rodents demonstrated that dopamine release was unnecessary for the oxygen and pH changes to occur.

The short term acidic shift followed by a return to base line in the monkey striatum during unpredicted reward (Figure 3) is strikingly similar to that seen upon unpredicted oral infusion of sucrose in rats (Roitman et al., 2008). Furthermore, the amplitude of the acidic shift following the reward in trials in which a cue (with some probability) previously predicted a juice reward was comparable to the shift during unpredicted reward (Figure 4B). This suggests that the increased metabolism and localized pH decrease, coupled with the accompanying blood flow changes, was similar at juice delivery in both trials in which a cue signaled its delivery (or not) and in unpredicted trials. Our responses for both oxygen changes (Figure 5) and pH changes (Figures 2 and 3) correlate well with fMRI results (Raichle, 1998) in which the signals provide an index of metabolic activity and changes in blood flow related to neural activity in the brain. Electrophysiological studies have shown neuronal activation in striatal neurons following unexpected rewards (Roitman et al., 2005), and unexpected rewards enhance striatal fMRI responses in humans (Berns et al., 2001). fMRI signals have three components: the initial dip, principal peak, and post-stimulus undershoot (Huettel et al., 2009). The physiological component of the principal peak is an increase in cerebral blood flow which would cause a transient alkaline shift (due to clearance of CO_2_). In predicted reward trials, time traces in the caudate nucleus obtained with fMRI show an increase in intensity at the beginning of a trial and at the presentation of the reward-predicting cue that continues for 1–3 s following reward delivery (Delgado et al., 2000; Zink et al., 2004; Haber and Knutson, 2010). The predominant pH response during predicted reward trials was remarkably similar with a short term basic shift upon cue presentation (Figure 2) whose amplitude tracked the probability of reward (Figure 4A). The strong similarity of the fMRI signals and the electrochemical pH responses occurs because both arise from an increase in striatal blood flow.

The second most predominant response of the trials where a cue predicted the probability of a reward was an acidic shift following the basic shift (Figures 2B and D). The physiological basis of this response is likely due to an increase in metabolism following neural activity. The time-course for this response aligns with the undershoot component of the fMRI signal (Frahm et al., 2008; Dechent et al., 2011). The peak amplitude of the pH shift in trials that showed this acidic shift was not different in rewarded and unrewarded trials, but did show a trend towards increasing with risk (Figure 4). However, approximately ~17% of responses showed an *initial acidic* pH shift following the cue presentation. Oxygen sensors (Thompson et al., 2003) and BOLD fMRI (Kim et al., 2000) have shown that there is a fast increase in oxygen consumption coinciding with neuronal activity followed by a delayed increase in oxygen levels. This initial dip is thought to be a better direct indicator of neuronal activity than the principal peak which may reflect increases in cerebral blow flow (Ances, 2004; Buxton et al., 2004). The initial dip does not always proceed the peak in fMRI studies and could account for the small percentage of *initial* acidic responses observed here (Blanchard et al., 2011).

In the *p* = 0.50 reward probability trials in animal 3 and in 14% of the free reward trials in animals 1 and 2, dopamine-like signals were obtained. Electrophysiology data show that dopamine neurons fire bursts following reward delivery (Robinson et al., 2008). The burst firing of dopamine neurons transiently increases concentrations in the terminal regions (Sombers et al., 2009). Here, the dopamine release during free reward was elevated for about one second, which is on the time scale previously reported for naturally occurring transient dopamine release (Wightman and Robinson, 2002; Robinson et al., 2003; Wightman et al., 2007), but that is more rapid than dopamine release during natural reward delivery in rats (Roitman et al., 2004). Most likely, this is due to the faster dopamine uptake rate in monkeys (Cragg et al., 2000, 2002). The oxidation and reduction peaks of the dopamine signal (Figures 3 and 5) match closely those for *in vitro* dopamine obtained in calibration sessions (Figure 1, *r*^2^ = 0.84). Similarly, the *in vivo* O_2_ signal (Figure 5) shows close agreement with that obtained *in vitro*. The cyclic voltammograms attributed to pH changes (Figure 2) also showed close correlation with those recorded under known conditions. Therefore, in some trials in which the risk was the lowest we were able to obtain an oxygen and dopamine-like signal that reflects previous reports using these data acquisition parameters.

The difference in distribution of blood vessels between rodent and primate brains may account for both the observed heterogeneity of responses and the masking of dopamine by the pH signal. The intercapillary distance in rhesus monkey cortex is ~70 μm, 50% larger than in rat cortex (Levin et al., 1976; Lamanna et al., 2004). The greater separation of capillaries would allow for greater pH gradients. However, cortical respiration rates in primates are lower than in rodents (Attwell and Laughlin, 2001), which may offset this affect to some degree. The small size of the carbon microelectrodes allows it to be placed closer or further way from a site of neural activity or a blood vessel during any given recording. Thus, an electrode placed closer to a site of neural activity would record acidic pH changes and an electrode placed closer to a blood vessel would likely measure alkaline pH changes more readily. Future studies with a blood flow sensor will further elucidate the link between neural activity, metabolism and blood flow.

### Conflict of interest statement

The authors declare that the research was conducted in the absence of any commercial or financial relationships that could be construed as a potential conflict of interest.
